# GPAT Gene Silencing in Muscle Reduces Diacylglycerols Content and Improves Insulin Action in Diet-Induced Insulin Resistance

**DOI:** 10.3390/ijms21197369

**Published:** 2020-10-06

**Authors:** Iwona Kojta, Piotr Zabielski, Kamila Roszczyc-Owsiejczuk, Monika Imierska, Emilia Sokołowska, Agnieszka Błachnio-Zabielska

**Affiliations:** 1 Department of Hygiene, Epidemiology and Metabolic Disorders, Medical University of Bialystok, 15-222 Bialystok, Poland; paszkiewicziwona89@gmail.com (I.K.); m.imierska@gmail.com (M.I.); emiliasokolowska.umwb@gmail.com (E.S.); 2 Department of Medical Biology, Medical University of Bialystok, 15-222 Bialystok, Poland; piotr.zabielski@umb.edu.pl (P.Z.); kamilar33@wp.pl (K.R.-O.)

**Keywords:** insulin resistance, skeletal muscle lipid metabolism, gene silencing, shRNA, electroporation

## Abstract

Skeletal muscle is an important tissue responsible for glucose and lipid metabolism. High-fat diet (HFD) consumption is associated with the accumulation of bioactive lipids: long chain acyl-CoA, diacylglycerols (DAG) and ceramides. This leads to impaired insulin signaling in skeletal muscle. There is little data on the involvement of DAG in the development of these disorders. Therefore, to clarify this enigma, the gene encoding glycerol-3-phosphate acyltransferase enzyme (GPAT, responsible for DAG synthesis) was silenced through shRNA interference in the gastrocnemius muscle of animals with diet-induced insulin resistance. This work shows that HFD induces insulin resistance, which is accompanied by an increase in the concentration of plasma fatty acids and the level of bioactive lipids in muscle. The increase in these lipids inhibits the insulin pathway and reduces muscle glucose uptake. GPAT silencing through electroporation with shRNA plasmid leads to a reduction in DAG and triacylglycerol (TAG) content, an increase in the activity of the insulin pathway and glucose uptake without a significant effect on ceramide content. This work clearly shows that DAG accumulation has a significant effect on the induction of muscle insulin resistance and that inhibition of DAG synthesis through GPAT modulation may be a potential target in the treatment of insulin resistance.

## 1. Introduction

High-fat diet (HFD) consumption and obesity are associated with intracellular lipid accumulation, which leads to various pathological conditions, e.g., insulin resistance and type 2 diabetes (T2D). The mechanism underlying the state has not yet been fully understood. Skeletal muscles play a key role in whole-body glucose homeostasis and therefore are a main place of insulin resistance. In obese and T2D subjects, plasma free fatty acid (FFA) concentration is often elevated compared to lean, healthy counterparts [1]. An increased concentration of FFA was observed in skeletal muscles in animals fed HFD [2,3,4,5,6,7], in obese people [8,9] and T2D patients [10]. Plasma FFAs enter muscle cells by diffusion or via protein transporters. To date, three groups of fatty acid transporters have been identified: fatty acid translocase (FAT/CD36) [11], fatty acid binding protein (FABPpm) [12] and fatty acid transport protein family (FATP1–6). After entering the cell, fatty acids attach coenzyme A in a reaction catalyzed by acyl-Co synthase (ACS), resulting in the formation of long chain acyl-CoA (LCA-CoA) [13]. LCA-CoA can be used as a substrate in de novo synthesis of other lipids, e.g., triacylglycerols (TAG), diacylglycerols (DAG), ceramides (Cer), or they can be directed towards mitochondrial β-oxidation [14]. A very important mediator in LCA-CoA transport across the mitochondrial membrane is carnitine palmitoyltransferase 1 (CPT1). This enzyme is responsible for the formation of acyl-carnitines by catalyzing the transfer of the acyl group from LCA-CoA to l-carnitine. However, the main intermediate of FA de novo synthesis, malonyl-CoA, is an intracellular inhibitor of CPT1 and, thus, also indirectly inhibits β-oxidation. Increased FFA uptake and reduced β-oxidation probably contribute to intramyocellular lipid (IMCL) accumulation and the development of insulin resistance. Although, initially, it was supposed that the accumulation of triacylglycerols is directly responsible for inducing changes in the insulin pathway, now, researchers’ attention is focused on the role of biologically active lipids in the inhibition of this pathway. There are three groups of biologically active lipids that are potentially involved in the induction of muscle insulin resistance: LCA-CoA, ceramides and DAG. The results of studies conducted so far have demonstrated that the level of all these lipid groups increases in insulin resistant muscles. Increased LCA-CoA content and impaired glucose uptake have been shown in obese people [15,16] and animals fed HFD [2,5]. It has been observed that muscle LCA-CoA accumulation affects glucose uptake through activation of protein kinase C (PKC) [17]. LCA-CoA also indirectly affects the function of the insulin pathway because it can be used as a substrate for the de novo synthesis of other bioactive lipids, such as diacylglycerols and ceramides. An increase in the intracellular ceramide content was observed in the muscles of obese, insulin-resistant Zucker rats [18], animals fed HFD [2,5] and obese insulin-resistant people [19]. Ceramide has been shown to inhibit the insulin pathway at the level of protein kinase B (Akt/PKB) by activating phosphatase A2 (PPA2), which maintains Akt/PKB in an unphosphorylated state [20]. Another group of biologically active lipids that inhibit the activity of the insulin pathway are DAGs. Cellular DAG can be generated as a result of phospholipid breakdown, or it can be synthesized de novo via the esterification of LCA-CoA to glycerol 3-phosphate [21]. Glycerol-3-phosphate acyltransferase (GPAT1) catalyzes the reaction of sn-glycerol-3-phosphate acylation at the sn-1 position to form lysophosphatidic acid (LPA). This enzyme is responsible for the key stage of de novo DAG synthesis. The accumulation of intracellular DAG has been demonstrated in the insulin-resistant muscle of HFD rats [2,5,22,23], Zucker rats [18] and obese insulin-resistant people [24]. In addition, threefold increases in intracellular DAG levels were observed in lipid-infused rats, which was associated with inhibition of the insulin pathway through PKCθ activation [25]. There is ongoing discussion about which of the above-mentioned lipid mediators plays the most important role in FA-induced skeletal muscle insulin resistance. Most of the work on the participation of lipids in the induction of muscle insulin resistance relates to ceramide, and much less is known about the effect of DAG accumulation on the insulin sensitivity of skeletal muscles. Therefore, the aim of this study was to elucidate the role of DAG in the induction of muscle insulin resistance. To achieve this goal, we studied the effect of in vivo shRNA-mediated GPAT gene silencing on bioactive lipid accumulation and the insulin signaling pathway in the gastrocnemius muscle of mice with diet-induced insulin resistance.

## 2. Results

### 2.1. Insulin Sensitivity

**Figure 1:**
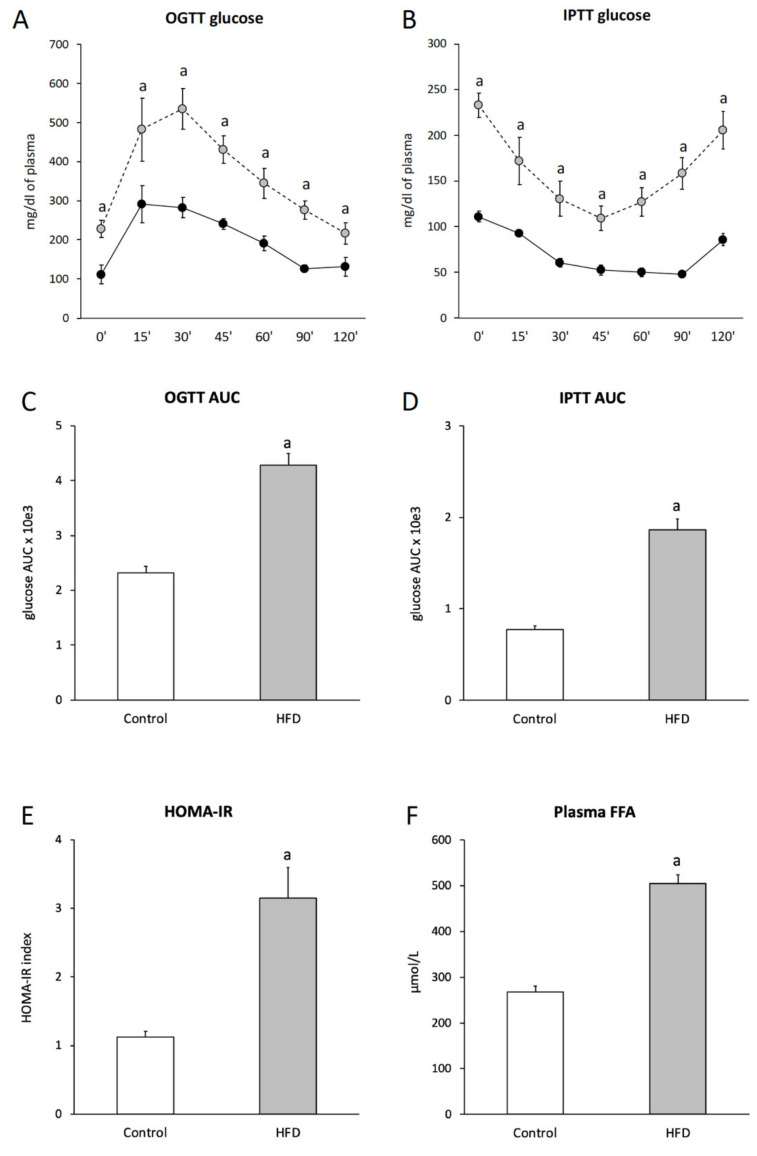
Impact of high-fat diet (HFD) consumption on plasma glucose and plasma fatty acid concentration. Panel **A**—plasma glucose profile during oral glucose tolerance test (OGTT); Panel **B**—plasma glucose profile during intraperitoneal insulin tolerance test (IPTT); Panel **C**—area under the plasma glucose curve for OGTT; Panel **D**—area under the plasma glucose curve for IPTT; Panel **E**—HOMA-IR value; Panel **F**—plasma free fatty acids (FFA). Values are mean ± SD (*n* = 8 per group); a —*p* < 0.05 vs. control; significance by *t*-test; HFD-High-fat diet.

HFD-fed mice developed insulin resistance, as evidenced by increased fasting glucose and insulin concentration, impaired glucose tolerance, decreased insulin responsiveness and an increase in the Homeostatic Model Assessment of Insulin Resistance (HOMA-IR)value, as compared to the control group (Figure 1; Table 1).

### 2.2. Plasma Free Fatty Acids (FFA) Concentration

Total plasma FFA concentration increased in the HFD group by 89% (*p* < 0.05), as compared to the control group. More than a twofold increase was observed in the following fatty acids: C14:0, C18:2, C18:1, C18:0, C20:4, C20:0, C22:0, C24:1 and C24:0 (*p* < 0.05) (Figure 1F; Table S1).

### 2.3. Fatty Acid Transporters

#### 2.3.1. CD36

**Figure 2:**
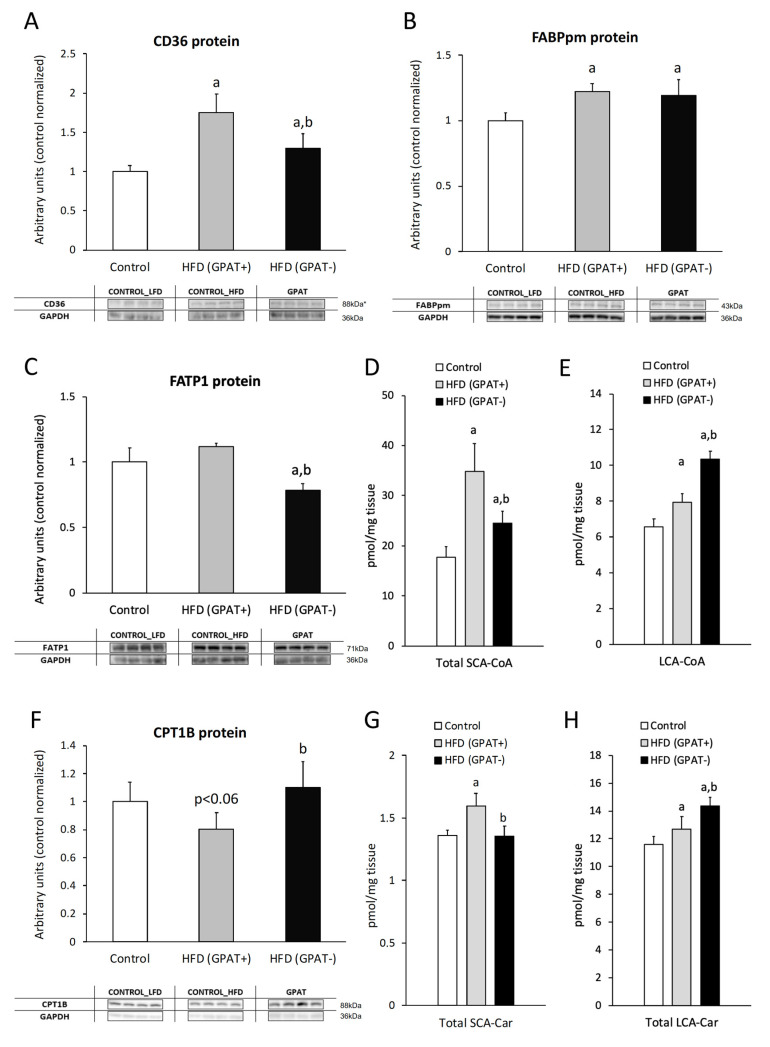
The impact of skeletal muscle GPAT silencing on the protein expression of fatty acid transporters, the content of acyl-CoA’s and acyl-carnitines in gastrocnemious muscle of high-fat diet mice. Panel **A**—protein expression of CD36; Panel **B**—protein expression of FABPpm; Panel **C**—protein expression of FATP1; Panel **D** and **E**—the content of short-chain acyl-CoA and long-chain acyl-CoA, respectively. Panel **F**—protein expression of CPT1B; Panel **G** and **H**—the content of short-chain and long-chain acyl-carnitines, respectively. Values are mean ± SD (*n* = 8 per group); a—*p* < 0.05 vs. control; b—*p* < 0.05 vs. HFD_(GPAT+)_; significance by ANOVA; HFD—high-fat diet. * The observed molecular weight of indicated proteins is different to the theoretical one, as stated by the antibody manufacturer.

The content of CD36 significantly increased in HFD_(+GPAT)_ skeletal muscle compared control muscle (*p* < 0.05). GPAT silencing significantly decreased CD36 in HFD_(−GPAT)_ muscle, as compared to HFD_(+GPAT)_ (*p* < 0.05), yet the values were significantly higher than in the control (Figure 2A). 

#### 2.3.2. FABPpm

The content of FABPpm in HFD_(+GPAT)_ skeletal muscle significantly increased compared to the control (*p* < 0.05). GPAT silencing did not alter increased FABPpm expression, and the protein content was still significantly higher in HFD_(−GPAT)_ muscle, as compared to the control (*p* < 0.05) (Figure 2B).

#### 2.3.3. FATP1

The FATP1 content in skeletal muscle significantly decreased in HFD_(-GPAT)_ muscle, as compared to both the control and HFD_(+GPAT)_ muscle (*p* < 0.05). Although the FATP1 content slightly increased in the HFD_(+GPAT)_ group, this change did not reach statistical significance (Figure 2C).

### 2.4. Skeletal Muscle Short and Long Chain Acyl-CoA

The total short-chain acyl-CoA (SCA-CoA) content significantly increased in HFD_(+GPAT)_ muscle, as compared to control muscle. GPAT silencing significantly decreased SCA-CoA in HFD_(-GPAT)_ muscle, yet the value was still higher than observed in control muscle. LCA-CoA content increased similarly in HFD_(+GPAT)_ muscle, yet GPAT silencing further elevated muscular LCA-CoA to a level significantly higher than both the control and HFD_(-GPAT)_ muscle. The highest increase in both muscle groups was noticed for C14:0-CoA, C18:2-CoA, C18:0-CoA and C22:0-CoA (*p* < 0.05 for all cases) (Figure 2D,E; Table S2).

### 2.5. Skeletal Muscle CPT1B Content.

The content of CPT1B decreased in the muscle of HFD_(+GPAT)_, as compared to the control, yet the value did not reach significance level. GPAT silencing increased CPT1B in HFD_(−GPAT)_ muscle to a level significantly higher than in HFD_(+GPAT)_ muscle (*p* < 0.05), reaching values similar to the control group (Figure 2F).

### 2.6. Skeletal Muscle Short and Long-Chain Acyl-Carnitine Content

The dynamics of skeletal muscle acyl-carnitines under HFD consumption displayed a similar pattern to the case of muscular acyl-CoA content. Short-chain acyl-carnitines significantly increased in HFD_(+GPAT)_ muscle, as compared to the control, and were normalized by GPAT silencing in HFD_(-GPAT)_ muscle (*p* < 0.05 in both cases). Long-chain species of acyl-carnitines increased in HFD_(+GPAT)_ muscle above the control values, and their content was further elevated by GPAT silencing in HFD_(-GPAT)_ muscle (*p* < 0.05 in both cases). In both cases the highest increase was noticed for C18:0-carnitine (Figure 2G,H; Table S3, Table S4).

### 2.7. Skeletal Muscle mRNA and Protein Content of GPAT 

**Figure 3:**
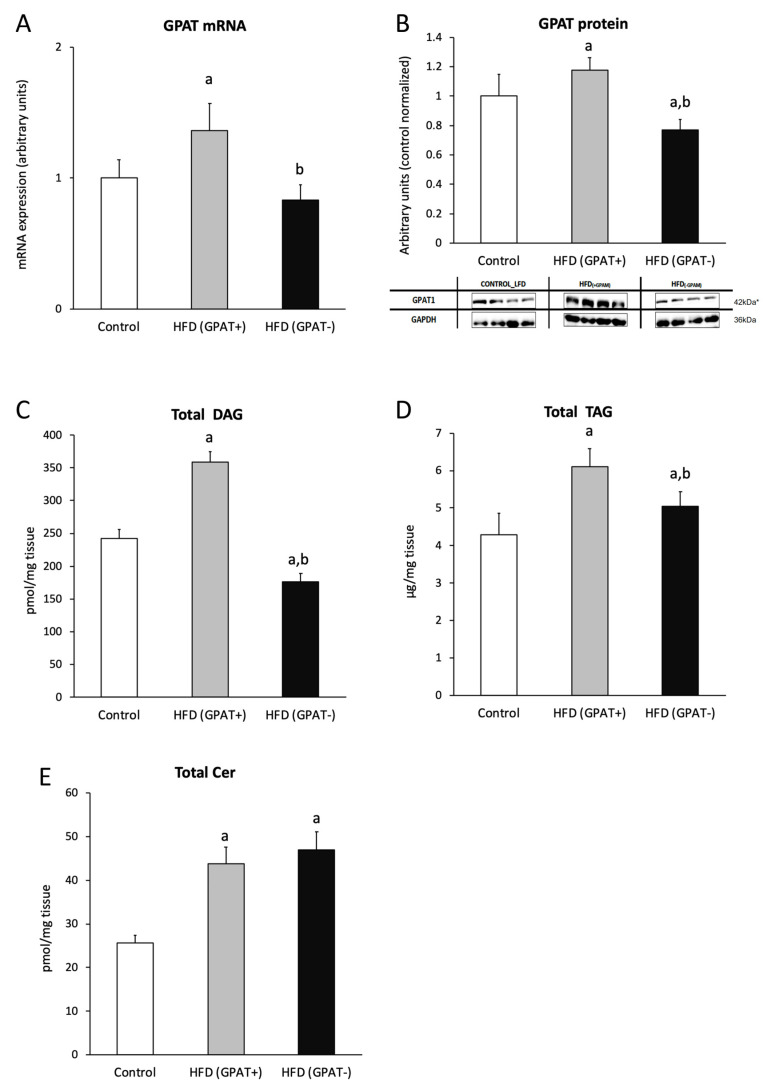
The impact of skeletal muscle GPAT silencing on the gene and protein expression of GPAT, and the content of bioactive lipids in gastrocnemious muscle of high-fat diet mice. Panel **A** and **B**—the expression skeletal muscle GPAT at the mRNA and protein level, respectively; Panel **C**—total content of diacylglycerols (DAG); Panel **D**—total content of triacylglycerols (TAG); Panel **E**—total content of ceramide (Cer). Values are mean ± SD (*n* = 8); a—*p* < 0.05 vs. control; b—*p* < 0.05 vs. HFD_(GPAT+)_; significance by ANOVA; HFD—high-fat diet. * The observed molecular weight of indicated proteins is different to the theoretical one, as stated by the antibody manufacturer.

Both, the mRNA and protein content of GPAT increased significantly in the HFD_(+GPAT)_ muscle of animals fed HFD, as compared to the control (*p* < 0.05), and decreased significantly in the HFD_(-GPAT)_ muscle, as compared to HFD_(+GPAT)_ (Figure 3A,B).

### 2.8. Skeletal Muscle DAG Level

Total content of DAG significantly increased in the HFD_(+GPAT)_ muscle, as compared to the control (*p* < 0.05). The highest increase was observed for C18:0/18:2, C16:0/18:0, C16:0/18:2 and 18:0/20:0. GPAT silencing decreased all the measured DAG species in the HFD_(-GPAT)_ muscle, as compared to both the control and HFD_(+GPAT)_ muscle (*p* < 0.05). The highest decrease was observed for C18:0/18:0, C18:2/18:2, C16:0/18:0 and C16:0/18:2 DAG species (*p* < 0.05 in all cases) (Figure 3C; Table 2).

### 2.9. Skeletal Muscle TAG Level

The content of triacylglycerols in skeletal muscle of HFD_(+GPAT)_ significantly increased, as compared to the control (*p* < 0.05), whereas, in the HFD_(-GPAT)_ muscle, the content of TAG significantly decreased, as compared to HFD_(+GPAT)_ (*p* < 0.05) (Figure 3D).

### 2.10. Skeletal Muscle Ceramide Content

Total ceramide content increased in HFD_(+GPAT)_ skeletal muscle under a high-fat diet and was not normalized under GPAT silencing in HFD_(-GPAT)_ muscle. The level of C16:0-Cer, C18:0-Cer, C20:0-Cer, C22:0-Cer, C24:1-Cer and C24:0-Cer significantly increased in the HFD_(+GPAT)_ muscle, as compared to the control. In the HFD_(-GPAT)_ muscle, a significant increase in the content of all measured sphingolipids was noted (*p* < 0.05 in all cases) (Figure 3E; Table S5).

### 2.11. Insulin Cascade Activation/Inhibition

#### 2.11.1. IR

**Figure 4:**
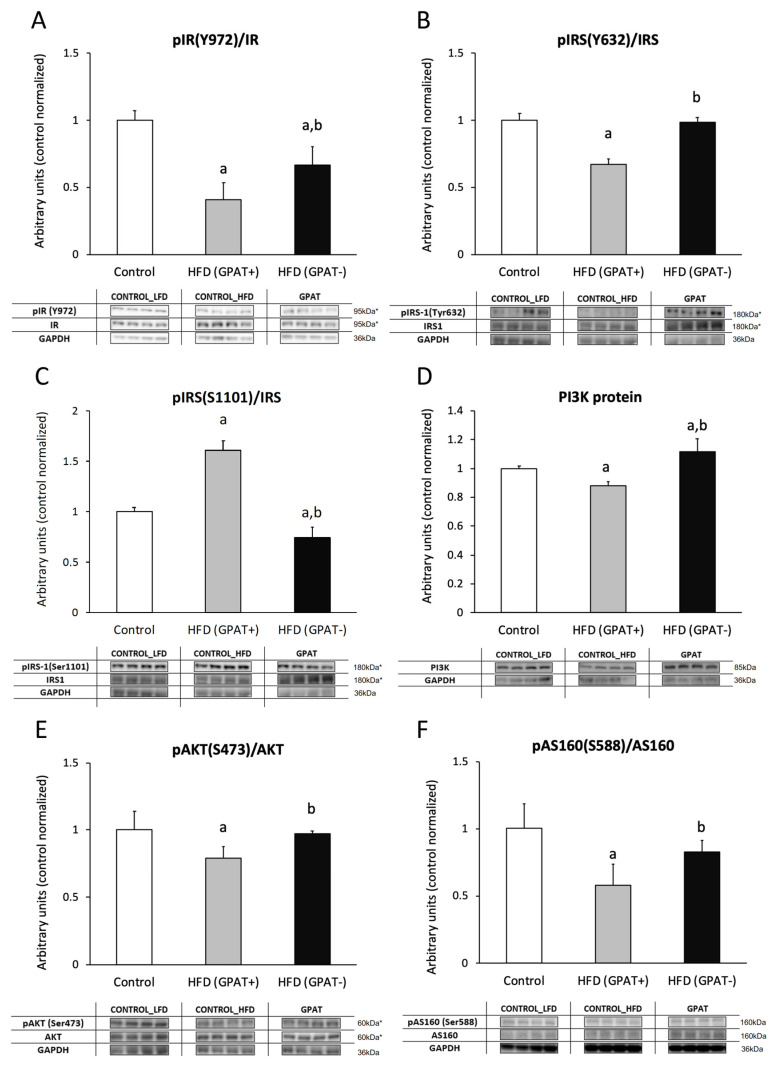
Activation of insulin signaling pathways in GPAT-silenced mouse gastrocnemious muscle. Panel **A**—insulin receptor phosphorylation (pIR Y972); Panel **B**—insulin receptor substrate 1 (IRS1) tyrosine phosphorylation (pIRS1 Y632); Panel **C**—IRS1 serine phosphorylation (pIRS S1101). Panel **D**—protein expression of phosphoinositide 3-kinase (PI3K); Panel **E**—serine phosphorylation of protein kinase B/Akt (pAKT S473); Panel **F**—serine phosphorylation of Akt/PKB 160kDa substrate (pAS160 S588). Values are mean ± SD (*n* = 8); a—*p* < 0.05 vs. control; b—*p* < 0.05 vs. HFD_(GPAT+)_: statistics by ANOVA; HFD—high-fat diet. * The observed molecular weight of indicated proteins is different to the theoretical one, as stated by the antibody manufacturer.

The phosphorylation state of the insulin receptor (IR) decreased significantly in the HFD_(+GPAT)_ muscle, as compared to the control (*p* < 0.05). GPAT silencing increased IR receptor phosphorylation in HFD_(-GPAT)_ muscle, as compared to HFD_(+GPAT)_ (*p* < 0.05), yet the level was still significantly lower than in the control (Figure 4A).

#### 2.11.2. IRS-1

The level of tyrosine phosphorylation of the insulin receptor substrate 1 (IRS1) protein decreased in HFD_(+GPAT)_ muscle, as compared to the control (*p* < 0.05), and it was normalized by GPAT silencing in HFD_(-GPAT)_ muscle. The level of inhibitory serine phosphorylation was significantly elevated in HFD_(+GPAT)_ muscle, as compared to the control (*p* < 0.05), and decreased in the HFD_(-GPAT)_ group, as compared to both the control and HFD_(+GPAT)_ muscle (*p* < 0.05) (Figure 4B,C).

#### 2.11.3. PI3K

The protein content of PI3K decreased in HFD_(+GPAT)_ muscle, as compared to the control (*p* < 0.05), and significantly elevated in HFD_(-GPAT)_ muscle, as compared to both the control and HFD_(+GPAT)_ (*p* < 0.05) (Figure 4D).

#### 2.11.4. Akt Phosphorylation

The ratio of pAkt(Ser473) to Akt significantly decreased in muscle of HFD_(+GPAT)_ mice, as compared to the control group (*p* < 0.05), and increased in HFD_(-GPAT)_, as compared to both the control and HFD_(+GPAT)_ (*p* < 0.05) (Figure 4E).

#### 2.11.5. AS160

The ratio of pAS160(Ser588)/AS160 significantly decreased in the HFD_(+GPAT)_ muscle, as compared to the control (*p* < 0.05). GPAT silencing normalized AS160 phosphorylation in HFD_(-GPAT)_ muscle to a level not different from the control (*p* < 0.05) (Figure 4F).

### 2.12. Glucotransporter 4 (GLUT4)

**Figure 5:**
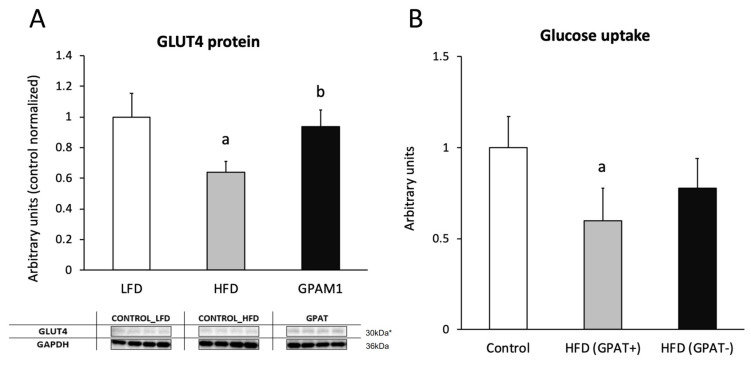
The impact of GPAT silencing on mouse gastrocnemious muscle insulin-stimulated glucose uptake. Panel **A**—protein expression of glucotransporter 4 (GLUT4); Panel **B**—insulin-stimulated glucose uptake. Values are mean ± SD (*n* = 8); a—*p* < 0.05 vs. control; b—*p* < 0.05 vs. HFD_(GPAT+)_; significance by ANOVA; HFD- high-fat diet. * The observed molecular weight of indicated proteins is different to the theoretical one, as stated by the antibody manufacturer.

The content of the GLUT4 protein significantly decreased in HFD_(+GPAT)_ skeletal muscle, as compared to the control (*p* < 0.05), and was normalized by GPAT silencing in HFD_(-GPAT)_ skeletal muscle (*p* < 0.05) (Figure 5A). 

### 2.13. Skeletal Muscle Glucose Uptake

Insulin-stimulated glucose uptake was significantly decreased in HFD_(+GPAT)_ muscle, as compared to the control (by 40%; *p* < 0.05). GPAT silencing in HFD_(-GPAT)_ muscle increased glucose uptake to values not significantly different from those observed in the control (Figure 5B).

## 3. Discussion

Skeletal muscle is one of the most important tissues responsible for glucose and lipid metabolism. There are many studies that show the relationship between the accumulation of bioactive lipids and impaired insulin signaling in skeletal muscle [2,5,15,16,17,18,19,20,24]. However, the contribution of individual lipid groups to the inhibition of insulin pathways is still unknown. The aim of this study was to elucidate the effect of HFD on the induction of muscle insulin pathway disorders and the role of DAG accumulation in this process. To achieve this goal, in mice fed HFD, we transfected the gastrocnemius muscle of one limb with GPAT gene shRNA, while the muscle of the other limb was transfected with scrambled shRNA. We found that HFD consumption led to insulin resistance, as evidenced by increased fasting glucose and insulin concentration, impaired glucose tolerance, reduced insulin responsiveness, an increase in HOMA-IR and decreased muscular glucose uptake compared to the control group. We have noticed a significant increase in plasma FFA concentration and a significant increase in all the studied lipid groups in nonsilenced muscle, which was in line with observations made by other research groups [1,2,3,4,5,7,19,26]. However, we wanted to find out how, under conditions of induced insulin resistance, reducing DAG content would affect the insulin pathway and whether it would improve muscle glucose uptake. In our study, in the HFD_(+GPAT)_ muscle, the content of FA transporters CD36 and FABPpm significantly increased, as compared to the control, and, in the muscles with HFD_(-GPAT)_, we observed a decrease in the level of all examined FA transporters compared to muscles with HFD_(+GPAT)_. The increased content of FA transporters in the skeletal muscles of HFD-fed or obese mice was also previously repeatedly presented by other researchers [27,28,29]. GPAT is an enzyme that participates in the synthesis of DAG and, because DAG is an intermediate in de novo TAG synthesis, GPAT also participates in the synthesis of TAG. The decrease in the level of FA transporters in muscles with HFD_(-GPAT)_ may be caused by a decrease in the capacity of these muscles for TAG synthesis in the presence of increased plasma FFA concentration. This may be an adaptation and protection against increased FFA transport to the cell in a situation of reduced ability to store fat in the form of TAG. 

Upon entering the cell, FAs are transformed into LCA-CoAs. In the HFD_(+GPAT)_ gastrocnemius, we observed an increased content of LCA-CoA, but an even higher increase was observed in the HFD_(-GPAT)_. This phenomenon may be due to reduced flux of LCA-CoAs in the direction of TAG synthesis. LCA-CoAs can be directed to mitochondrial β-oxidation or be used as a substrate for the de novo synthesis of other lipids. For LCA-CoAs that are directed for β-oxidation through the mitochondrial carnitine shuttle, carnitine palmitoyltransferase 1 (CPT1) catalyzes the conversion of cytoplasmic LCA-CoA into acylcarnitines. In the present work, despite the observed decrease in CPT1 content in the HFD_(+GPAT)_ muscles, we observed simultaneous increases in the content of both the long- and short-chain acyl-carnitines, which suggests the saturation of mitochondrial β-oxidation capacity. Interestingly, GPAT silencing, despite normalized CPT1 protein expression, led to an increase in both long-chain acyl-carnitine and LCA-CoA content and a decrease in their short-chain counterparts, which suggests augmentation of β-oxidation in silenced muscle. The observed changes in the content of intramuscular lipids in the HFD_(-GPAT)_ muscle, especially the decrease in the level of DAG and the increase in acyl-carnitine, positively affect the functioning of the insulin pathway. DAGs are well-known cellular second messengers that activate protein kinase C. Phosphorylation of tyrosine residues at the IRS1 is necessary for the proper functioning of the insulin pathway, whereas phosphorylation of serine/threonine residues inhibits this pathway. Accumulation of DAG in muscles has been found to activate certain PKC isoforms that are responsible for the phosphorylation of serine residues in IRS, which inhibits insulin signaling [30,31,32]. In the present work, we observed that, in the HFD_(-GPAT)_ muscles, the activity of the insulin pathway increases, which was evident in the content and degree of the phosphorylation of individual proteins of this pathway. Moreover, we showed that, in the HFD_(-GPAT)_ muscles, the degree of phosphorylation of the IR at Tyr 972 and the IRS1 at Tyr632 increases compared to the HFD_(+GPAT)_ muscles. At the same time, we found a decrease in the phosphorylation of Ser1101 in IRS1. It has previously been demonstrated that an increase in DAG content correlates with a decrease in the functioning of the insulin pathway at the IR and IRS levels [31,33]. However, our work is the first in which we have shown, within the muscles of a living organism, how silencing of the gene encoding the enzyme responsible for de novo DAG synthesis affects the functioning of the insulin pathway in comparison to the HFD_(+GPAT)_ muscle of the same animal with induced insulin resistance. Moreover, a similar effect to DAG on the insulin pathway is attributed to LCA-CoA; however, although accumulation of LCA-CoA in HFD_(-GPAT)_ muscle was observed, the activity of the insulin pathway improved, suggesting that LCA-CoA does not play the dominant role in determining the insulin pathway disorder and that a decrease in DAG content plays a more important role. LCA-CoA accumulation led to the increase in other lipids, namely, ceramide. Interestingly, gene silencing of GPAT did not affect muscle ceramide content, which points to DAG as the culprit, at least in part, for fat-induced muscle insulin resistance. However, data from ob/ob mice lacking the GPAT1 gene (ob/ob-Gpat1 ^- / -^ mice) showed that, although the content of hepatic TAG and DAG was significantly lower than that of the ob/ob mice, liver insulin signaling was not improved [34].

Another insulin pathway protein we studied in our work was PI3K. We found that, in the HFD_(+GPAT)_ muscles, we observed only a slight decrease in the content of this protein, while, in HFD_(-GPAT)_ muscles, an increase in the level of this protein was noted. This shows that silencing the GPAT gene and decreasing DAG production enhances the action of the insulin pathway at this stage as well. Many works on muscle insulin resistance relate to the role of ceramide accumulation in inducing insulin pathway disorders. Available data indicate that ceramide accumulation inhibits the insulin pathway at the Akt/PKB level [2,5,19,35,36]. In this study, we observed a significant decrease in Akt and AS160 phosphorylation in the HFD_(+GPAT)_ muscles, while, in the HFD_(-GPAT)_ muscles, despite the lack of a statistically significant change in ceramide content, we observed an increase in Akt and AS160 phosphorylation. This is probably the effect of increased activation of the insulin pathway at a higher level of IRS1. Furthermore, in addition to the observed improvement in the functioning of the insulin pathway in the HFD_(-GPAT)_ muscles, we also observed an increase in the GLUT4 level. All changes in the insulin pathway observed in our studies due to inhibition of DAG production lead to partial improvement in glucose uptake by these muscles compared to HFD_(+GPAT)_ muscles. Interestingly, overall amelioration of insulin resistance at the level of insulin signaling cascade was more pronounced than the improvement in muscular glucose uptake. This discrepancy could arise from other effects of prolonged HFD consumption, namely, the negative impact of lipid overabundance on overall muscle energy metabolism. By triggering sarcoplasmic reticulum stress, mitochondrial dysfunction and mitochondria-related oxidative stress, intracellular lipids could decrease the metabolic ability to oxidize both the fatty acids and carbohydrates despite improvement in the insulin signaling cascade.

## 4. Materials and Methods 

The study was approved by the Local Ethical Committee for Animal Experiments of the Medical University of Olsztyn, Poland (approval number 43/2016). The experiments were carried out on male C57BL/6 mice (20 g) (Jackson Laboratory, Bar Harbor, ME, USA). Animals were housed in standard conditions (21 °C ± 2, 12 h light/12 h dark cycle) with free access to water and food pellets. The animals were randomly divided into the following groups: a control group of mice (*n* = 8), fed ad libitum a standard rodent diet, with the both hindlimb gastrocnemius muscles treated with scrambled shRNA plasmid and HFD group (*n* = 8), and mice fed HFD, with one hindlimb gastrocnemius muscle (HFD_(-GPAT)_) electroporated with shRNA plasmids targeted towards GPAT1 (the enzyme responsible for de novo DAG synthesis) and the opposed hindlimb gastrocnemius muscle (HFD_(+GPAT)_) electroporated with scrambled shRNA plasmid. All mice were fed for 8 weeks with an appropriate diet. The standard rodent diet contained 70% carbohydrate, 10% fat and 20% protein (% energy) (D12450J, Research Diets INC, New Brunswick, NJ, USA). HFD were composed of 20% carbohydrate, 60% fat and 20% protein (% energy). In HFD, saturated fatty acids were derived from lard (D12492). 

Two weeks before the sacrifice, an oral glucose tolerance test (OGTT) was performed, followed one week later by an insulin tolerance test (IPTT). Precisely 20 min before the euthanasia, an intravenous bolus of 2-deoxy-[1,2-3H (N)]-d-glucose (Perkin Elmer, Waltham, MA, USA) was given through a lateral tail vein injection, followed by an intraperitoneal injection of 0.5 U/Kg insulin (NovoRapid, Novo Nordisk A/S, Bagsværd, Denmark) to measure muscle glucose uptake and insulin-stimulated protein phosphorylation cascade. The mice were euthanized by cervical dislocation. Immediately thereafter, the gastrocnemius muscles were taken and frozen in liquid nitrogen and then stored at −80 °C until analysis. 

### 4.1. Plasmids and In Vivo Electroporation

**Figure 6:**
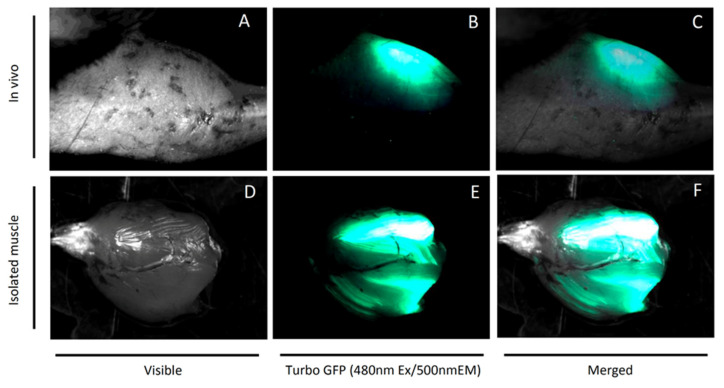
Green fluorescent protein (TurboGFP) reporter gene expression in mouse muscle at 6 weeks after electroporation-mediated plasmid transfection. Panel **A**—visible light photo of mouse hindlimb; Panel **B**—transcutaneous Turbo-GFP fluorescence of electroporated gastrocnemius and tibialis anterior muscle; Panel **C**–**A** and **B** merged; Panel **D**—visible light photo of isolated mouse gastrocnemius muscle; Panel **E**—Turbo-GFP fluorescence of electroporated gastrocnemius; Panel **F**–**D** and **E** merged. Fluorescence stereomicroscopy performed with Nightsea SFA-RB-GO fluorescence adapter/DeltaPix Invenio 5SIII CMOS camera.

Bacterial stocks producing shRNA/Turbo green fluorescent protein (TurboGFP) plasmids or shRNA scrambled sequences through an mCMV promoter were purchased from Dharmacon (currently Horizon Discovery, Cambridge, UK). After bacterial culture, according to manufacturer guidelines, plasmids were isolated with the GeneJET Plasmid Maxiprep Kit (Thermo Scientific, Waltham, MA, USA) and stored in −80 °C. A plasmid mixture encoding three different target shRNA sequences was prepared in 150 mM PBS (pH = 7.2) prior to injection. After 2 weeks of acclimatization and HFD feeding, the animals were anesthetized in an induction chamber with the use of ~2% isoflurane in oxygen with UNO BV rodent anesthesia system (UNO, Zevenaar, The Netherlands). Mice were kept on heating blanket under general isoflurane anesthesia for the entire electroporation procedure. The area above the gastrocnemius muscle was shaved and 30 µL of hyaluronidase (0.4 U/µL hyaluronidase in sterile Tyrode) was injected in two divided doses of 15 µL from two sides of the muscle. Two hours later, 40 µL (from 2 µg/µL stock) of plasmid solution was slowly injected into the gastrocnemius muscle using a 27-gauge needle. Electric pulses (175 volts/cm and eight pulses at 200 ms intervals) were applied by a pair of stainless steel electrode plates (1 cm^2^ area) placed on each side of the isolated muscle belly with the use of BTX ECM 830 Electroporation Generator (Holliston, MA, USA) [37]. Another leg was injected accordingly with the plasmid containing scrambled shRNA sequences. Expression of the GFP reporter gene in both legs was monitored transcutaneously weekly with the use of a UV flashlight. Exemplary GFP fluorescence of an isolated gastrocnemius muscle is presented in Figure 6.

### 4.2. Lipid Measurements

#### 4.2.1. Plasma FFA

Plasma FFA concentration was measured by high-performance liquid chromatography/tandem mass spectrometry (UHPLC/MS/MS) according to Persson et al. [38]. Briefly, to each plasma sample, a known amount of C14:0-d27, C15:0, C16:0-d31, C17:0, C18:1-d9 and C18:0-d35 (Avanti Polar Lipids, Alabaster, AL, USA) was added as an internal standard prior to extraction with freshly prepared Dole solution (isopropanol: heptane:1 M H_2_SO_4_ (40:10:1; *v*/*v*/*v*)). The extracts were evaporated under a nitrogen stream. The dried samples were resuspended in buffer A for the LC/MS analysis. Fatty acids were separated on the LC using a reverse-phase Zorbax SB-C18 column 2.1 × 150 mm, 1.8 µm (Agilent Technologies, Santa Clara, CA, USA), using a two-buffer system. Buffer A was 80% acetonitrile and 0.5 mM ammonium acetate; buffer B was 99% acetonitrile and 1% 0.5 mM ammonium acetate. The concentrations of FFA were measured against a six-point standard curve prepared with albumin-conjugated FFA chemical standards.

#### 4.2.2. Sphingolipids

The content of sphingolipids was measured using a UHPLC/MS/MS approach according to Blachnio-Zabielska et al. [39], with minor modifications. Briefly, the skeletal muscle samples (~20 mg) were pulverized and homogenized in 0.25 M sucrose, 25 mM KCl, 50 mM Tris and 0.5 mM EDTA, pH = 7.4. Immediately afterwards, the internal standard solution (Sph-d7, SPA-d7, S1P-d7, C15:0-d7-Cer, C16:0 -d7-Cer, C18:1-d7-Cer, C18:0-d7-Cer, 17C/20:0-Cer, C24:1-d7-Cer and C24-d7-Cer, Avanti Polar Lipids, Alabaster, AL, USA) as well as the extraction mixture (isopropanol:water:ethyl acetate, 30:10:60; v:v:v) was added to each homogenate. The mixture was vortexed, sonicated and centrifuged. The supernatant was transferred to a new tube and the pellet was reextracted. Both supernatants were combined and evaporated under a nitrogen stream. The dried sample was reconstituted in LC Solvent B (2 mM ammonium formate and 0.1% formic acid in methanol) for UHPLC/MS/MS analysis. Sphingolipids were analyzed with a Sciex QTRAP 6500+ triple quadrupole mass spectrometer (AB Sciex Germany GmbH, Darmstadt, Germany) using a positive ion electrospray ionization (ESI) source (except for S1P, which was analyzed in the negative mode) with multiple reaction monitoring (MRM) against standard curves constructed for each compound. The chromatographic separation was performed using an ultraperformance liquid chromatograph (Shimadzu Nexera X2 UHPLC, Shimadzu Corporation, Kyoto, Japan). The analytical column was a reverse-phase Zorbax SB-C8 column 2.1 × 150 mm, 1.8 μm (Agilent Technologies, Santa Clara, CA, USA). Chromatographic separation was conducted in binary gradient using 1 mM ammonium formate and 0.1% formic acid in water as solvent A, and 2 mM ammonium formate and 0.1% formic acid in methanol as solvent B at the flow rate of 0.4 mL/min.

#### 4.2.3. Diacylglycerols

The content of DAG was measured using a UPLC/MS/MS approach according to Blachnio-Zabielska et al. [40]. Diacylglycerols were extracted together with sphingolipids. A known amount of internal standard mix (Deuterated DAG Mixture I and Mixture II, Avanti Polar Lipids, Alabaster, AL, USA) was added prior to lipid extraction. Next, samples were extracted as described above. The following DAG were quantified: C18:1/18:2, C16:0/18:2, C16:0/16:0, C16:0/18:1, C18:0/20:0, C18:0/18:1, C18:1/18:1, C18:0/18:2 and C16:0/18:0. This was done using a triple quadrupole mass spectrometer (Sciex QTRAP 6500+ AB Sciex Germany GmbH, Darmstadt, Germany) and a positive ion electrospray ionization (ESI) source with multiple reaction monitoring (MRM) against the concentration standard curves prepared for each compound.

#### 4.2.4. Malonyl-CoA and Long-Chain Acyl-CoA

LCA-CoA concentration was measured according to Blachnio-Zabielska et al. [41]. Briefly, malonyl-CoA as well as LCA-CoA were extracted according to Minkler et al. [42]. Prior to the extraction procedure, a known amount of C15:0-CoA, 16:0(d4) CoA, C17-CoA, C19:0-CoA, C21:0-CoA, C23:0:-CoA and 24:0(d4) CoA (Avanti Polar Lipids, Alabaster, AL, USA) was added as an internal standard. The molecules (C14:0-CoA, C16:0-CoA, C16:1-CoA, C18:2-CoA, C18:1-CoA, C18:0-CoA, C20:0-CoA, C22:0-CoA, C24:1-CoA and C24:0-CoA, Avanti Polar Lipids, Alabaster, AL, USA) were separated on a reverse-phase Agilent ZORBAX Extend-C18 Column, 2.1 × 150 mm, using a binary gradient with ammonium hydroxide (NH_4_OH) in water and NH_4_OH in ACN. The LCA-CoA concentration was quantified using multiple reaction monitoring (MRM) on a triple quadrupole mass spectrometer (Sciex QTRAP 6500+, AB Sciex Germany GmbH, Darmstadt, Germany) in positive electrospray ionization (ESI) mode against the concentration standard curves prepared for each compound.

#### 4.2.5. Acyl-carnitines

Acyl-carnitine concentration was measured according to Giesbertz [43] with a small modification. In short, skeletal muscle samples were pulverized and then extracted with the use of ice-cold methanol and centrifuged at 10,000 *g*, 4 °C, for 10 min. The supernatant was transferred to a fresh tube and dried under nitrogen. Prior to extraction, an internal standard (C17-carnitine) was added to each sample. Acyl-carnitines were derivatized to their butyl esters by incubation with 100 µL n-butanol containing 5% *v*/*v* acetyl chloride at 60 °C for 20 min with shaking (800 rpm, Eppendorf Thermomixer Comfort, Eppendorf, Hamburg, Germany). Subsequently, samples were evaporated to dryness, reconstituted in 100 µl methanol/water and transferred to glass vials for UHPLC/MS/MS analyses. Acyl-carnitines were quantified on a Sciex QTRAP 6500+ triple quadrupole mass spectrometer (AB Sciex Germany GmbH, Darmstadt, Germany) using positive ion electrospray ionization (ESI) with multiple reaction monitoring (MRM) against standard curves constructed for each compound. The chromatographic separation was performed using ultraperformance liquid chromatography (Shimadzu Nexera-X2 UHPLC). The analytical column was a reverse-phase Zorbax SB-C18 column 2.1 × 150 mm, 1.8 μm (Agilent Technologies, Santa Clara, CA, USA). 

#### 4.2.6. Triacylglycerols

The content of intramuscular TAG was measured by the use of the High Sensitivity Triglyceride Fluorometric Assay Kit (MAK264-1KT, Merck KGaA, Darmstadt, Germany) according to the manufacturer’s protocol.

### 4.3. Western Blotting 

Tissue samples were homogenized in RIPA buffer (Merck KGaA, Darmstadt, Germany) supplemented with 0.5 mM tris(2-carboxyethyl)phosphine (TCEP, reducing agent, Merck KGaA, Darmstadt, Germany) and protease and phosphatase inhibitors. After denaturation in Laemmli buffer, proteins were separated by SDS-PAGE (AnykD Criterion TGX gels and a Criterion Cell electrophoresis cell, BioRad, Hercules, CA, USA) and transferred on PVDF membrane (BioRad Trans Blot SD semidry transfer cell with discontinuous buffer system (Tris/CAPS/15% methanol for anode and Tris/CAPS 0.1% SDS for cathode). The membrane was probed with the appropriate primary antibody. The following target proteins were quantified using rabbit primary antibodies: glucose transporter 4 (GLUT4) (Abcam, Cambridge, MA, USA), CD36 (Novus Biologicals, Centennial, CO, USA), FATP1 (Novus Biologicals, Centennial, CO, USA), FABPpm, creatinine-O-palmitoyltransferase 1 (CPT1) (Abcam, Cambridge, MA, USA), GPAT1/GPAM, (Abcam, Cambridge, MA, USA), Akt, pAktSer473, IRS1, pIRS1(Tyr632), pIRS1(Ser1101), IR, pIR(Y972), AS160, AS160Ser588 (Cell Signalling Technology, Danvers, MA, USA) and glyceraldehyde 3-phosphate dehydrogenase (GAPDH) (Abcam, Cambridge, MA, USA). The binding of the primary antibody was detected after incubation with HRP-conjugated secondary antibody, Clarity™ Western ECL chemiluminescent substrate (Bio-Rad), and visualized using the Bio-Rad ChemiDoc XRS+ imaging system. Band intensities were quantified with the use of the Bio-Rad Image Lab software package |(Software version 6.1). Values were normalized to GAPDH protein expression measured from parallel runs and expressed as fold changes over control group values. Unless stated otherwise, all chemicals and equipment used for immunoblotting were purchased from Bio-Rad.

### 4.4. Real-Time PCR 

Total RNA was extracted from muscle tissue using the mirVana Isolation Kit (ThermoScientific, Waltham, MA, USA) according the manufacture’s protocol. The RNA was reverse transcribed into cDNA using the Transcriptor First Strand cDNA Synthesis Kit (Roche, Mannheim, Germany). Real-time PCR was performed with RealTime ready Custom Assays for GPAT and GAPDH, which was used as a housekeeping gene using a LightCycler480 system (Roche, Mannheim, Germany). The results were normalized to GAPDH expression measured in each sample.

### 4.5. Insulin-Stimulated Glucose Uptake

Insulin-stimulated glucose uptake was measured with the use of 2-deoxy-[1,2-3H (N)]-D-glucose according to Fueger et al. [44]. Briefly, 20 min prior to euthanasia, radiolabeled glucose was injected as a bolus through the lateral tail vein (bolus of 12 µCi/25 g animal, a total of 25 µL, Perkin-Elmer, Waltham, MA), followed by 0.5 U of insulin per Kg of fat-free mass, given intraperitoneally. To estimate plasma 2-deoxy-[1,2-3H (N)]-d-glucose enrichment, blood samples (25 μL were collected at 2.5, 5, 7.5, 10, 12.5 and 15 min after putting the radiotracer bolus into the heparinized tube, immediately centrifuged and stored in -80 °C until analysis. Simultaneously, plasma glucose was measured with the use of an AccuCheck Aviva glucometer (Roche, Mannheim, Germany). Plasma and tissue 2-deoxy-[1,2-3H (N)]-d-glucose specific activity were measured with the use of 0.3 N Ba(OH)_2_/0.3 N ZnSO_4_ precipitation and a scintillation counter. Tissue-incorporated radioactive tracer was liberated by sonication in 6% HClO_4_, neutralization with 5 M KOH and fractionation into 2-deoxy-D-glucose and 2-deoxy-d-glucose-6-phosphate with 0.3 N Ba(OH)_2_/0.3 N ZnSO_4_. Plasma samples were deproteinized with 0.3 N Ba(OH)_2_/0.3N ZnSO_4_. Radioactivity was measured with a Packard Tri-Carb 1900TR liquid scintillation counter (Perkin Elmer, Waltham, MA). Tissue-incorporated 2-deoxyglucose-6-phosphate was calculated as the difference in radioactivity in the HClO_4_ and Ba(OH)_2_/ZnSO_4_ tissue extracts. Insulin-stimulated glucose uptake was calculated using the following equation:

(1) $$\mathrm{Rg}\;=\;\frac{\mathrm{Cm}*\left(T\right)}{\int_{0}^{T}\left(\mathrm{Cp}*/\mathrm{Cp}\right)}\;*\Delta t$$

where Cm* (T) is the tissue radioactivity of 2-deoxy-[1,2-3H (N)]-d-glucose/mg at the end of the experiment (DPM/mg), ∆t is the time from glucose bolus injection to tissue collection (min) and ∫T,0 (Cp∗/Cp) is the area under the plasma glucose enrichment curve calculated with the use of the trapezoidal rule (DPM/mg/min).

**Figure 7:**
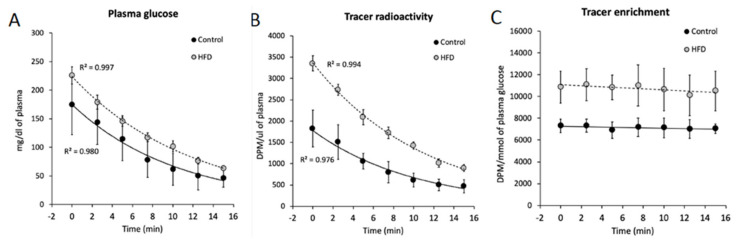
Plasma glucose and 2-deoxy-[1,2-3H (N)]-D-glucose profiles during 0.5U/Kg intraperitoneal insulin challenge. Panel **A**—plasma glucose concentration; Panel **B**—plasma 2-deoxy-[1,2-3H (N)]-d-glucose tracer radioactivity; Panel **C**—plasma glucose tracer enrichment after intravascular tracer and insulin bolus injection. Values are mean ± SD; *n* = 8 per group.

Exemplary plasma glucose concentration and 2-deoxy-[1,2-3H (N)]-d-glucose radioactivity curves after bolus injection of the tracer and insulin are presented in Figure 7.

### 4.6. Plasma Insulin and Glucose Concentration

Plasma glucose was determined using an AccuChek Aviva glucometer (Roche, Mannheim, Germany). Plasma insulin was measured with an ELISA insulin assay (Rat/Mouse Insulin Millipore, Merck KGaA, Darmstadt, Germany). 

### 4.7. Oral Glucose Tolerance Test (OGTT)

OGTT in fasted animals was performed as follows: blood glucose was measured 15, 30, 60, 90 and 120 min after oral glucose administration in a dose of 2 g/Kg. Blood samples from the tail vein were measured by using glucometer AccuChek Aviva (Roche, Mannheim, Germany). At 15 and 60 min, blood was also collected for insulin concentration measurement. The area under the glucose concentration curve was measured with the use of the trapezoidal rule.

### 4.8. Insulin Tolerance Test (ITT)

Fasted animals received an intraperitoneal injection of insulin in a dose of 0.75 U/Kg. Glucose concentration was measured on samples obtained from the tail vein using the glucometer AccuChek Aviva (Roche, Mannheim, Germany) at 0, 15, 30, 45, 60, 90 and 120 min after insulin injection. The area under the glucose concentration curve was measured with the use of the trapezoidal rule.

### 4.9. HOMA-IR (Homeostatic Model Assessment of Insulin Resistance)

The HOMA-IR index value was calculated according to formula [45]:

HOMA-IR = [fasting glucose (mg/dL) × fasting insulin (μIU/mL)]/2430

### 4.10. Protein Concentration

Protein content in homogenates was measured with the Pierce 660 nm protein assay kit (Thermo Fisher Scientific, Waltham, MA, U.S.). Bovine serum albumin (fatty acid free) was used as a standard. 

### 4.11. Statistical Analysis

Results were expressed as mean +/− standard deviation. Significant differences between the control, low-fat diet (LFD), non-silenced high-fat diet HFD (GPAT+) and silenced, high-fat diet HFD (GPAT−) sample groups were identified by ANOVA with Tukey Honest Significant Difference post hoc test (Tukey HSD) for equal group size. The differences in whole-body anthropometric values between the LFD and HFD groups were identified with unpaired Student’s *t*-test. The significance threshold was set at *p* < 0.05 in both cases.

## 5. Conclusions

Taken together, our work is the first in which we comprehensively studied the role of GPAT-derived DAG in the induction of insulin pathway dysfunction and reduction of glucose uptake in skeletal muscle. We showed that the HFD consumption induced insulin resistance, which was accompanied by an increased plasma FFA concentration and an increase in the lipid level for LCA-CoA, ceramides, DAG, TAG and acyl-carnitines in skeletal muscles. The observed increase in lipids induced an inhibition of the insulin pathway, as evidenced by a decrease in the level and/or degree of protein phosphorylation in the insulin pathway, which results in a reduction in muscle glucose uptake. On the other hand, in the HFD(-GPAT) muscles, a decrease in DAG and TAG levels was observed, which increased the activity of the insulin pathway and, in turn, partially improved glucose uptake. Our research has clearly shown that the accumulation of DAG has a significant impact on the induction of muscle insulin resistance and that inhibition of DAG synthesis may be a potential target in the therapy of insulin resistance.

## Figures and Tables

**Table 1:** Glucose metabolism parameters in the plasma of mice fed a control and high-fat diet.

	Control	HFD
Glucose (mg/dL)	109 ± 14	184 ± 22 ^a^
Insulin (μU/mL)	25.1 ± 2.9	41.3 ± 2.2 ^a^
OGTT AUC (×10^3^)	2.32 ± 0.12	4.27 ± 0.21 ^a^
ITT AUC (×10^3^)	0.78 ± 0.04	1.86 ± 0.12 ^a^
HOMA-IR	1.12 ± 0.09	3.14 ± 0.45 ^a^

Values are mean ± SD; *n* = 8 per group; a—*p* < 0.05 vs. Control; significance by *t*-test.

**Table 2:** The impact of GPAT silencing on the content of diacylglycerol molecular species in mouse gastrocnemius muscle.

	16:0/16:0	16:0/18:0	16:0/18:1	16:0/18:2	18:0/18:0	18:0/18:1	18:0/18:2	18:1/18:1	18:2/18:2	18:0/20:0
Control	13.41 ± 1.87	83.47 ± 11.87	32.18 ± 4.97	38.09 ± 5.91	2.10 ± 0.23	21.88 ± 3.11	0.96 ± 0.19	20.98 ± 2.31	24.91 ± 2.63	3.85 ± 0.58
HFD_(+GPAT)_	16.15 ± 2.47 ^a^	144.52 ± 16.79 ^a^	39.36 ± 4.67 ^a^	66.52 ± 11.60 ^a^	2.92 ± 0.23 ^a^	22.90 ± 3.65	1.98 ± 0.33 ^a^	23.37 ± 2.21 ^a^	34.00 ± 5.15 ^a^	7.23 ± 0.96 ^a^
HFD_(-GPAT)_	11.52 ± 1.11 ^b^	59.86 ± 10.31 ^a,b^	24.15 ± 1.21 ^a,b^	21.31 ± 3.68 ^a,b^	0.91 ± 0.11 ^a,b^	16.74 ± 2.42 ^a,b^	1.53 ± 0.13 ^a,b^	16.73 ± 1.25 ^a,b^	18.68 ± 1.72 ^a,b^	4.90 ± 0.50 ^a,b^

Values are mean pmol/mg of tissue ± SD; *n* = 8 per group; a—*p* < 0.05 vs. control; b—*p* < 0.05 vs. HFD_(+GPAT)_; significance by ANOVA; HFD—high-fat diet.

## Supplementary Materials

Supplementary Materials can be found at https://www.mdpi.com/1422-0067/21/19/7369/s1.

## Abbreviations

HFD	High-fat diet
GPAT	Glycerol-3-phosphate acyltransferase
DAG	Diacylglycerols
T2D	Type 2 diabetes
FFA	Free fatty acids
FA	Fatty acids
FAT/CD36	Fatty acid translocase
FABPpm	Fatty acid binding protein
FATP1–6	Fatty acid transport protein family
ACS	Acyl-Co synthase
LCA-CoA	Long chain acyl-CoA
TAG	Triacylglycerols
Cer	Ceramides
CPT1	Carnitine palmitoyltransferase 1
IMCL	Intramyocellular lipids
PKC	Protein kinase C
Akt/PKB	Protein kinase B
PPA2	Phosphatase A2
LC/MS	Liquid chromatography mass spectrometry
HOMA-IR	Homeostatic model assessment
