# Supplementary material for: GPAT Gene Silencing in Muscle Reduces Diacylglycerols Content and Improves Insulin Action in Diet-Induced Insulin Resistance
Source: Int J Mol Sci. 2020 Oct 6;21(19):7369. doi: 10.3390/ijms21197369 (PMC7583033; doi:10.3390/ijms21197369)
Supplement: Supplementary file 1 [file ijms-21-07369-s001.pdf]

Supplementary Materials

Table S1. Individual plasma free fatty acids concentration in mice fed control and high-fat diet.

	C14:0	C16:0	C16:1	C18:0	C18:1	C18:2	C20:0	C20:2	C20:4	C22:0	C22:6	C24:0	C24:1
**Control**	7.08±1.46	70.66±8.03	5.99±1.39	30.02±2.39	69.23±7.29	61.29±11.03	1.09±0.20	2.32±0.37	9.57±1.44	3.90±0.31	1.67±0.20	4.04±1.04	0.16±0.02
**HFD**	16.81±1.09[a]	95.14±10.59[a]	5.27±0.39	60.99±3.99[a]	141.35±8.55[a]	137.72±6.58[a]	3.97±0.30[a]	2.52±0.27	19.58±1.00[a]	11.19±0.69[a]	2.19±0.17[a]	8.14±0.64[a]	0.36±0.06[a]

Values are mean µmol/l of plasma ± SD; $n = 8$ per group; a – $p < 0.05$ vs Control

Table S2. Impact of GPAT gene silencing on the content of individual acyl-CoA's in mouse gastrocnemius muscle.

	**C2:0**	**C3:0**	**C4:0**	**C14:0**	**C16:0**	**C16:1**
**Control**	1.15±0.17	16.59±2.26	0.016±0.004	0.12±0.01	0.85±0.10	0.64±0.06
**HFD$_{(+GPAT)}$**	1.69±0.25[a]	33.14±5.34[a]	0.028±0.003[a]	0.20±0.02[a]	0.99±0.13[a]	0.68±0.07
**HFD$_{(-GPAT)}$**	2.18±0.19[a,b]	22.20±2.68[a,b]	0.040±0.006[a,b]	0.26±0.03[a,b]	1.15±0.10[a,b]	0.79±0.04[a,b]

**C18:0**	**C18:1**	**C18:2**	**C20:0**	**C22:0**	**C24:0**	**C24:1**
0.73±0.09	2.59±0.33	1.54±0.18	0.017±0.003	0.022±0.004	0.028±0.004	0.013±0.002
1.20±0.15[a]	2.80±0.34	1.96±0.27[a]	0.019±0.004	0.033±0.005[a]	0.031±0.005	0.009±0.001
1.73±0.09[a,b]	3.37±0.38[a,b]	2.91±0.34[a,b]	0.028±0.004[a,b]	0.039±0.008[a,b]	0.039±0.007[a,b]	0.012±0.001[b]

Values are mean pmol/mg of tissue ± SD; $n = 8$ per group; a – $p < 0.05$ vs Control; b – $p < 0.05$ vs HFD$_{(+GPAT)}$.

Table S3. Impact of GPAT gene silencing on the level of individual short-chain acyl-carnitines in mouse gastrocnemius muscle.

	C2	C3	C4	C5	C5DC	C6	C8	C10	C12
**Control**	48.14±5.39	3.16±0.34	3.82±0.48	2.41±0.33	0.015±0.0024	0.79±0.09	0.24±0.05	0.17±0.03	0.26±0.02
**$HFD_{(+GPAT)}$**	55.04±6.19[a]	3.80±0.40[a]	4.42±0.46[a]	2.80±0.37[a]	0.018±0.0020[a]	0.95±0.16[a]	0.28±0.03[a]	0.21±0.04[a]	0.31±0.03[a]
**$HFD_{(-GPAT)}$**	45.44±5.21[b]	3.42±0.24[b]	3.63±0.27[b]	2.31±0.40[b]	0.016±0.0022	0.81±0.11[b]	0.21±0.03[b]	0.17±0.04	0.23±0.03[a,b]

Values are mean pmol/mg of tissue ± SD; *n* = 8 per group; a – $p < 0.05$ vs Control; b – $p < 0.05$ vs $HFD_{(+GPAT)}$.

Table S4. Impact of GPAT gene silencing on the level of individual long-chain acyl-carnitines in mouse gastrocnemius muscle.

	**C14**	**C16**	**C18**	**C18:1**
**Control**	1.47±0.20	2.46±0.24	0.72±0.10	6.94±0.59
**HFD$_{(+GPAT)}$**	1.19±0.18[a]	2.93±0.33[a]	1.23±0.14[a]	7.31±0.51
**HFD$_{(-GPAT)}$**	1.28±0.20	3.33±0.37[a,b]	1.36±0.07[a,b]	8.40±0.47[a,b]

Values are mean pmol/mg of tissue ± SD; *n* = 8 per group; a – $p < 0.05$ vs Control; b – $p < 0.05$ vs HFD$_{(+GPAT)}$.

Table S5. The impact of GPAT silencing on the content of ceramide molecular species in mouse gastrocnemious muscle.

	d18:1/C14:0	d18:1/C16:0	d18:1/C18:0	d18:1/C18:1	d18:1/C20:0	d18:1/C22:0	d18:1/C24:0	d18:1/C24:1
**Control**	0.029±0.003	1.94±0.24	16.93±1.53	0.56±0.09	0.27±0.04	0.77±0.13	1.24±0.16	3.83±0.44
**HFD$_{(+GPAT)}$**	0.034±0.008	3.20±0.35[a]	30.27±3.42[a]	0.67±0.05[a]	0.57±0.10[a]	1.45±0.21[a]	2.55±0.32[a]	5.08±0.59[a]
**HFD$_{(-GPAT)}$**	0.041±0.004[a,b]	3.43±0.30[a]	32.62±4.15[a]	0.71±0.06[a]	0.66±0.13[a]	1.55±0.25[a]	2.68±0.19[a]	5.19±0.58[a]

Values are mean pmol/mg of tissue +/- SD; n=8 per group; a – p<0.05 vs Control; b – p<0.05 vs HFD$_{(+GPAT)}$.
